# RESILIENCE THROUGH CONNECTION: SOCIAL SUPPORT AND ELDER ABUSE, DISASTER, BEREAVEMENT, AND COMBAT

**DOI:** 10.1093/geroni/igz038.1581

**Published:** 2019-11-08

**Authors:** Ron Acierno, Wendy Muzzy

**Affiliations:** Medical University of South Carolina, Charleston, South Carolina, United States

## Abstract

Elder abuse prevalence among community residing adults is 10%, but this prevalence is cut by more than half among those who report high levels of social connection. Relatedly, elder abuse outcomes are significant, producing increased prevalence of anxiety and depressive disorders, the prevalences of which are, again, halved when one experiences abuse in the context of high social support. Similarly, mental health effects of natural disaster on older adults are virtually eliminated in the presence of high social support. Moreover, treatment for anxiety and depressive disorders is improved when high social support is present. We will present findings from five of our major studies in the aforementioned areas that underscore this point.

